# Keratin14 mRNA expression in human pneumocytes during quiescence, repair and disease

**DOI:** 10.1371/journal.pone.0172130

**Published:** 2017-02-15

**Authors:** Marco Confalonieri, Emanuele Buratti, Gabriele Grassi, Rossana Bussani, Marco Chilosi, Rossella Farra, Michela Abrami, Cristiana Stuani, Francesco Salton, Miriam Ficial, Paola Confalonieri, Lorenzo Zandonà, Maurizio Romano

**Affiliations:** 1 Pulmonology Department, University Hospital of Cattinara, Trieste, Italy; 2 Molecular Pathology, International Centre for Genetic Engineering and Biotechnology, Trieste, Italy; 3 Department of Life Sciences, University of Trieste, Trieste, Italy; 4 Institute of Pathologic Anatomy, University of Trieste, Trieste, Italy; 5 Department of Diagnostic and Public Health, Pathology Unit, University of Verona, Verona, Italy; 6 Department of Engineering and Architecture, University of Trieste, Trieste, Italy; Indian Institute of Toxicology Research, INDIA

## Abstract

The lung alveoli slowly self-renew pneumocytes, but their facultative regeneration capacity is rapidly efficient after an injury, so fibrosis infrequently occurs. We recently observed Keratin 14 (KRT14) expression during diffuse alveolar damage (DAD), but not in controls. We wonder if KRT14 may be a marker of pneumocyte transition from quiescence to regeneration. Quantitative PCR and Western blot analyses highlighted the presence of KRT14 (mRNA and protein) only in human lung samples with DAD or interstitial lung disease (ILD). In the exponentially growing cell lines A549 and H441, the mRNA and protein levels of KRT14 peaked at day one after cell seeding and decreased at day two, opposite to what observed for the proliferation marker E2F1. The inverse relation of KRT14 versus E2F1 expression holds true also for other proliferative markers, such as cyclin E1 and cyclin D1. Of interest, we also found that E2F1 silencing caused cell cycle arrest and increased KRT14 expression, whilst E2F1 stimulation induced cell cycle progression and decreased KRT14. KRT14 also increased in proliferative pneumocytes (HPAEpiC) just before transdifferentiation. Overall, our results suggest that KRT14 is a viable biomarker of pneumocyte activation, and repair/regeneration. The involvement of KRT14 in regenerative process may suggest a novel pharmaceutical target to accelerate lung repair.

## Introduction

The lung has a facultative regeneration ability: its reparative capacity is highly efficient, but the differentiated cells re-enter the cell cycle only when needed [1]. Moreover, fibrosis after lung injury may occur infrequently when this regenerative potential is upset or reduced [1]. In particular, ARDS (acute respiratory distress syndrome) with its pathological correlate DAD (diffuse alveolar damage) may serve as a natural model for lung injury-repair, subsequent remodelling/disease, or regeneration and function recovery. In fact, while 40–60% of patients die, surviving ARDS patients may experience pulmonary fibrosis or effective lung regeneration with complete functional recovery after 1 year or less from the acute episode. The progenitor cells for lung alveoli are the alveolar epithelial cells type 2 (pneumocytes type 2), while DN-p63+/KRT14+ basal cells are progenitors for the airways [2]. After lung injury, type 2 pneumocytes proliferate and transdifferentiate into alveolar epithelial cells type 1 (pneumocytes type 1). Unfortunately, this process is not usually reported by pathologists for the lack of effective biomarkers. In a recent immunohistochemical study in human lung biopsy/autopsy samples, we showed that lung alveoli express keratin 14 (KRT14) in DAD/ARDS [3]. Interestingly, this factor was never found to be expressed in normal lung during progenitor cell quiescence. Therefore, the purpose of the present study was to verify if KRT14 could be a distinct molecular marker of adult alveolar cells (pneumocytes) transition from quiescence to regeneration. We assessed KRT14 mRNA expression and protein levels not only in human fresh lung samples from patients/controls, but also in primary isolated pneumocytes and cultured human pneumocyte cell lines under different experimental conditions.

## Materials and methods

### Subjects

Lung samples were obtained from 14 subjects (10 males), mostly were autopsies (9 cases) and the remnants were surgical biopsies for diagnostic purpose.

The lung samples were distributed among three groups: 6 normal lungs, 4 ARDS, and 4 interstitial lung diseases (ILD). Tissue samples were immediately frozen after surgery and stored at −80°C until use. Either samples from biopsy and autopsy were obtained during routine clinical procedures performed according to the local standard guidelines and regulations for diagnostic biopsy and autopsy. Written informed consent was obtained from all the biopsied patients or the patients' next of kin in case of autopsy. This study was approved by the institutional review board of the University of Trieste (ref. #48/2013).

### Cells and cell culture

Human pulmonary alveolar epithelial cells (HPAEpiC) were purchased from ScienCell Research Laboratories (Cat. No. 3200, Carlsbad, CA, USA). The HPAEpiC cells were isolated from human lung tissue and characterized by ScienCell using immunostaining for specific markers. According to the manufacturer’s specifications, they are only human alveolar type 2 epithelial cells (pneumocytes) [4, 5]. The human pulmonary alveolar epithelial cells were cultured in the basal medium supplemented with growth factors according to the manufacturer’ instruction in T25 culture flasks coated with poly-L-lysine (2μg/cm^2^). The culture medium, Alveolar Epithelial Cell Medium (ScienCell, #3201), was prepared by addition of 2% Fetal Bovine Serum (FBS, ScienCell, #0010), 1% Epithelial Cell Growth supplement (EpiCGS 100x, ScienCell, # 4152) and 1% of antibiotics (P/S 100x, 10000 units/mL. Penicillin—10000 μg/mL Streptomycin, ScienCell, # 0503). The HPAEpiC phenotype was verified at our Lab by immunocytochemistry with primary antibodies (DakoCytomation K0355, Denmark) and a chromogen (diaminobenzidine 0.05%, Sigma Aldrich, Missouri USA) to detect ABCA3 and PhmTOR, that are specific biomarkers of alveolar epithelial cells type 2.

The human lung adenocarcinoma cell lines H441 *(ATCC-CCL-185*, *Manassas*, *VA*, *USA*) and A549 (*ATCC-HTB-174*, *Manassas*, *VA*, *USA*) were chosen as acceptable models of lung surfactant-producing cells, were used as acceptable models of lung surfactant-producing cells, representative of human lung alveolar epithelium [6]. H441 was cultured in RPMI medium, A549 was cultured in Dulbecco's modified Eagle's high glucose medium DMEM (Euroclone, Celbio, Devon, UK). All media contained 10% fetal bovine serum (FBS), 2 mM L-glutamine, 100 U/ml penicillin and 100 μg/ml streptomycin (Euroclone, Celbio, Devon, UK). We evaluated the mRNA levels of KRT14 in H441 and A549 cell lines maintained under optimal growth conditions. To study the KRT14/cell proliferation ratio, we evaluated in parallel the mRNA levels of E2F1, a known promoter of cell proliferation [7].

#### Real time PCR

RNA from lung samples: total RNA was extracted from lung samples with Trifast reagent (Euroclone, Milan, Italy), according to manufacturer’s instruction. Reverse transcription was performed with Moloney murine leukaemia virus (MMLV) Reverse Transcriptase (Gibco-BRL, Life Technologies Inc., Frederick, MD, USA) and exameric random primers, according to manufacturer’s protocol. In order to detect any genomic DNA contamination, parallel reactions for each RNA sample were performed without MMLV. Quantitative PCRs were performed on a CFX96 real-time PCR detection system with iQ SYBR Green Supermix (Bio-Rad, Hercules, CA, USA). Primer sequences (5’-3’) are the following:

GAPDH_F: AAGGTGAAGGTCGGAGTCAA;
GAPDH_R: AATGAAGGGGTCATTGATGG;
KRT14 Ex1_F: GGCCTGCTGAGATCAAAGACTAC;
KRT14_Ex2_R: CACTGTGGCTGTGAGAATCTTGTT;
SFTPA1_Ex5_F: AGCCACACTCCACGACTTTAG; SFTPA1_Ex6_R:GGATTCCTTGGGACAGCAATG;
E2F1 (74 bp) F: CCAGGAAAAGGTGTGAAATC;
E2F1 (74 bp) R AAGCGCTTGGTGGTCAGATT
Cyclin E1 (112 bp) F: TGCCTGTACTGAGCTGGGCA;
Cyclin E1 (112 bp) R: GGCTGCAGAAGAGGGTGTTG;
CyclinD1 (70 bp) F: CCGTCCATGCGGAAGATC,
CyclinD1 (70 bp) R, 5’CCTCCTCCTCGCACTTCTGT.

The detailed qPCR conditions comprised 95°C for 3 min, followed by 45 cycles of 95°C for 15 sec and 60°C for 30 sec. The relative expression levels were calculated according to 2^ΔCt method, by using the equation ΔC_t_ = C_t(target)_−C_t(housekeeping)_ for Ct normalization. ΔCt (KRT14-RPL13a) values of ARDS patients and controls were used to create box plots of the real-time PCR measurements. The results represent the average and standard deviation of three independent experiments, with two technical replicates.

RNA from cell lines: total RNA was extracted from cell lines, quantified and the quality evaluated as previously described [8, 9]. Reverse transcription was performed using 1μg of total RNA in the presence of random hexamers and MuLV reverse transcriptase (Applera Co., USA). The primers (MWG Biotech, GA, 300 nM) and the real-time amplification conditions were as described [10, 11]. The relative amounts of each target mRNA were normalized by 28S rRNA content. In the case of HPAEpiC, surfactant protein A1 (SFTPA1) and KRT14 were quantitated by qPCR using the following program: 95°C for 3 min, followed by 45 cycles of 95°C for 15 sec and 60°C for 30 sec.

#### siRNA and plasmid transfections

The sequence of the siRNA (Eurogentec S.A., Belgium) directed against E2F1 mRNAs, has been previously reported [8]. The pool of three distinct siRNAs directed against KRT14 mRNA, was purchased by Dharmacon, (siGenome Smart Pool cat.no. M010602-01). The day before transfection, H441 cells were seeded at a density of 3.8 × 10^3^ cells/cm^2^ in 6 well plates in the presence of 3 ml of 10% fetal calf serum-containing medium. Transfections were performed using either the siRNAGL2 control siRNA (siGL2) or the specific siRNAs (siE2F1/siKRT14). Optimal transfection conditions were obtained by using Lipofectamine 2000 (1 mg/ml, Invitrogen) at a weight ratio siRNA-transfectant of 1:1. The mixture Liposome–siRNA (200 and 100 nM for siE2F1 and siKRT14, respectively) was then administered to the cells for 3 h at 37°C in the presence of serum-free medium. Afterwards, transfection medium was removed, cells were washed with 3 ml of PBS and then 4 ml of complete medium were added to the cells. E2F1 and enhanced green fluorescence protein (EGFP) expression plasmids (pE2F1 and pEGFP, respectively), were previously described [8, 9]. Optimal transfection conditions were set as reported [10]. Briefly, transfection mixture containing Lipofectamine 2000 (1 mg/ml, Invitrogen) and 2 μg of plasmidic DNA (weight ratio plasmid/transfectant of 1:2.5) was administered to 2.5 × 10^5^ cells in six well plates for 4h at 37°C in the presence of Optimem. Thereafter, the transfection medium was removed and replaced with growth medium. Target mRNA over-expression was observed at least for three days after transfection.

#### Western blot analysis

Protein extracts, separated by SDS-PAGE and transferred onto nitrocellulose membranes (Whatman, Clifton, NJ, USA), were probed with antibodies against KRT-14 (1:1000, ProteinTech, Chicago, IL). The following antibodies have been used: rabbit anti-cyclin D1 (0.4 μg/ml) and rabbit anti-GAPDH (0.2 μg /ml) were purchased from Santa Cruz Biotechnology (Santa Cruz, California, USA). The antibodies anti-KRT14, and mouse anti-E2F1 (5 μg /ml) were purchased from BD Bioscience Pharmingen (San Jose, California, USA). After incubation, the corresponding anti-rabbit or anti-mouse secondary HRP-conjugated antibody was used (Santa Cruz Biotechnology) and the blots developed using an enhanced chemiluminescent substrate (Supersignal West Pico, Pierce, Rockford, IL).

### Statistical analysis

P values were calculated by the GraphPad InStat tools (GraphPad Software, Inc., La Jolla, CA, USA) using the unpaired t-test with Bonferroni correction and the Mann-Whitney Test, as appropriate. P values < 0.05 were considered statistically significant.

## Results

### Expression of KRT14 in normal and diseased human lung samples

**Fig 1:**
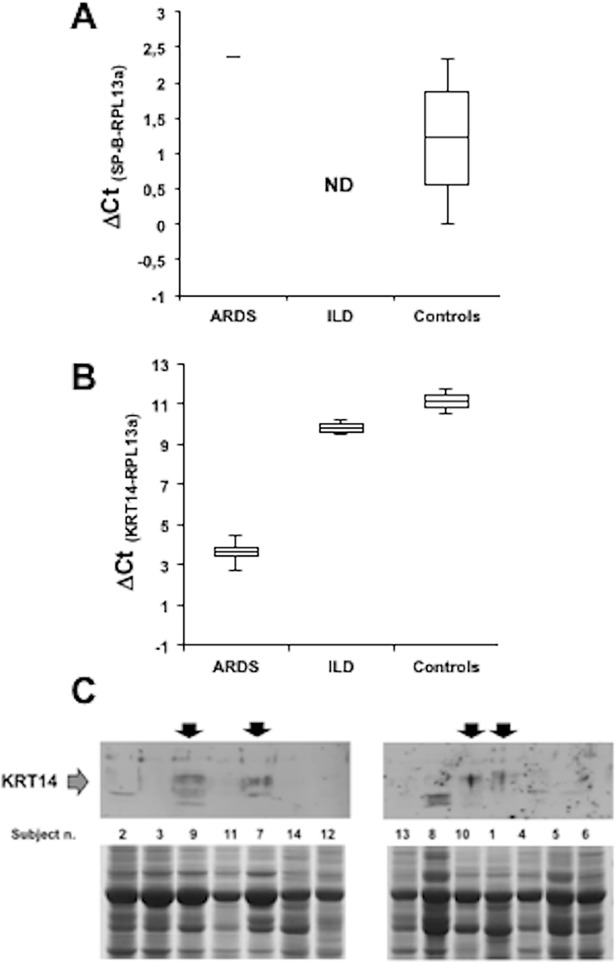
Expression of KRT14 in ARDS, ILD and normal lungs. A) Assessment of Surfactant-B (SP-B) expression at mRNA level. Box plots of the real-time PCR measurements as ΔCt (SP—B-RLP13a) values of ARDS patients (n = 4). ILD patients (n = 4) and Controls (n = 6). The box whisker plots visualize the minimum (end of the bottom whisker), the first quartile (bottom border of the box), the median (line through the box), the third quartile (top border of the box), and the maximum (end of the top whisker) of the distribution. DAD = diffuse alveolar damage, ILD = interstitial lung disease. B) Assessment of KRT14 expression at mRNA level. Box plots of the real-time PCR measurements as ΔCt (KRT14-RPL13a) values of ARDS patients (n = 4), ILD patients (n = 4) and Controls (n = 6). ΔCt values are shown in Table 1. The box whisker plots visualize the minimum (end of the bottom whisker), the first quartile (bottom border of the box), the median (line through the box), the third quartile (top border of the box), and the maximum (end of top whisker) of the distribution. C) Assessment of KRT14 expression at protein level. The immunoreactive material with MW compatible with that of KRT14 protein only in DAD patients. Upper panel: Western blot analysis. Lower panel: Ponceau red staining. The loading order is indicated in between the two panels and the Subject numbers correspond to that shown in Table 1.

The evaluation of KRT14 expression was performed on the lung samples from DAD, ILD, and control subjects (see Table 1 for subject characteristics). Surfactant-B (SP-B) plays a critical role in the functioning of healthy lungs and its impairment is commonly associated to acute respiratory distress syndrome (ARDS) [12]. Initially, we tried to correlate the severity of ARDS with the expression of SP-B, whose levels are known to be downregulated in the acute inflammatory lung diseases [12]. SP-B expression (tested by qPCR) was detected in 5 (out of 6) controls, in 1 (out of 6) DAD and in none of ILD patients (Fig 1A). These findings further support the hypothesis that a reduced SP-B expression is associated with severe lung dysfunction conditions, such as ARDS [12]. In parallel, we performed quantification of KRT14 expression both at mRNA and protein levels. Our patient population data show that in DAD (ARDS) patients, the calculated ΔCt (Ct_KRT14_ –Ct_RPL13a_) values were low but positive and were ranging between 2.76 and 4.45. On the contrary, the expression of KRT14 mRNA among the controls was always undetectable in 4 normal lung samples. Finally, the ΔCt values were >10 (11.78 and 10.46) in two ILD samples, denoting negligible expression level compared to ARDS patients (Fig 1B). Considering that the magnitude of the ΔCt values is inversely proportional to the expression level, these results confirmed that KRT14 is actively expressed mainly in DAD patients, even if some patient with ILD may also express it at low level. Consistent with these results, a Western blot analysis performed on the same lung specimens confirmed the presence of immunoreactive material with MW compatible with that of KRT14 only in DAD lung samples (Fig 1C).

**Table 1:** List of controls and patients screened for Keratin 14 (KRT14) expression.

Subject	Sex	Age	Lung histology	RPL13a	KRT14	ΔCt (KRT14- RPL13a)±SD
**1**	**M**	**79**	**Normal**	**+**	**-**	**nd**
**2**	**M**	**18**	**Normal**	**+**	**+**	**11.78±0.7**
**3**	**M**	**103**	**Normal**	**+**	**+**	**10.46±0.6**
**4**	**F**	**74**	**Normal**	**+**	**-**	**nd**
**5**	**F**	**92**	**Normal**	**+**	**-**	**nd**
**6**	**F**	**87**	**Normal**	**+**	**-**	**nd**
**7**	**M**	**54**	**ARDS**	**+**	**+**	**3.69±0.1**
**8**	**M**	**86**	**ARDS**	**+**	**+**	**4.47±0.16**
**9**	**M**	**49**	**ARDS**	**+**	**+**	**2.76±0.22**
**10**	**F**	**86**	**ARDS**	**+**	**+**	**3.65±0.27**
**11**	**M**	**65**	**ILD**	+	+	**10.22±0.15**
**12**	**M**	**99**	**ILD**	+	-	**nd**
**13**	**M**	**73**	**ILD**	+	-	**nd**
**14**	**M**	**86**	**ILD**	+	+	**9.44±0.18**

### KRT14 expression in proliferative human type II alveolar epithelial cells

**Fig 2:**
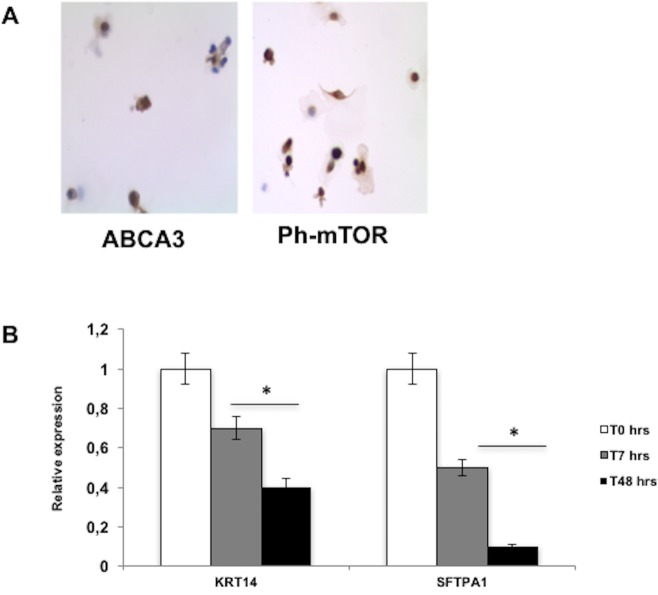
ABCA3/Ph-TOR staining in HPAEpiC and relative expression of KRT14/SFTPA. A) Immunocytochemistry staining in human HPAEpiC, showing that, at T = 0, the cells are ABCA3 and Ph-TOR positive. B) The mRNA expression of genes of interest, KRT14 and Surfactant protein A1 (SFTPA1) (type 2 pneumocyte marker), was monitored just after thawing (T0), after 7 hours (T7), and after 48 hours (T48). The normalization was carried out by using GAPDH and RPL13a housekeeping genes; data are reported as mean ± SD, n = 3, *p<0.05 compared to T0.

Immunocytochemistry staining carried out with the human HPAEpiC, comprised of only freshly cultured type 2 pneumocytes, showed that, at T = 0, these cells are ABCA3+ and Ph-TOR+ cells (Fig 2A), and suggests that HPAEpic cells have a proliferative alveolar epithelial cells type 2 phenotype, just before plating. Gene expression analysis at mRNA level monitored by Real Time PCR showed a drop of KRT14 within 48 h, after thawing (Fig 2B). During the same time slot, a decrease in the expression of the type 2 pneumocyte marker Surfactant Protein A1 (SFTPA1) was also monitored (Fig 2B). Even from a morphologic point of view, activated pneumocytes type 2 begin to change their shape before transdifferentiation from hyperplastic phenotype at baseline (T = 0) to a non-proliferating phenotype just after 24–48 hours from plating. In conclusion, these results suggest that the expression of KRT14 progressively decreases as transdifferentiation of pneumocytes type 2 towards type 1 starts.

### Expression of KRT14 in cultured H441 and A549 human pulmonary epithelial cells and its relationship with cell proliferation

To further explore the link between KRT14 expression with cell proliferation, we evaluated KRT14/cell proliferation relation in two *in-vitro* models of human pneumocytes, namely H441 and A549 cell lines. To study the KRT14/cell growth relation, we evaluated KRT14 expression levels and cell number; we also included the evaluation of the mRNA levels of E2F1, cyclin E1 and cyclin D1, known promoters of cell proliferation interconnected by a positive feed-back loop [11].

**Fig 3:**
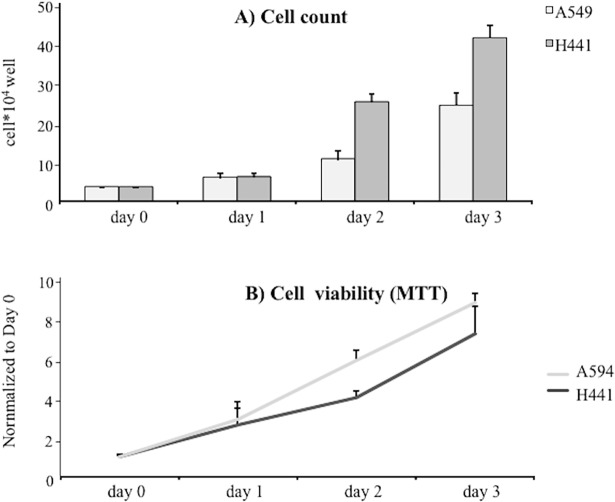
Kinetic of human pulmonary cell lines A549 and H441 growth. A) Cell count of A549 and H441 lines at day 0, 1, 2, and 3 in basal growth conditions. B) Cell viability of A549 and H441 lines at day 0, 1, 2, and 3 in basal growth conditions. All data are reported as mean ± SD.

**Fig 4:**
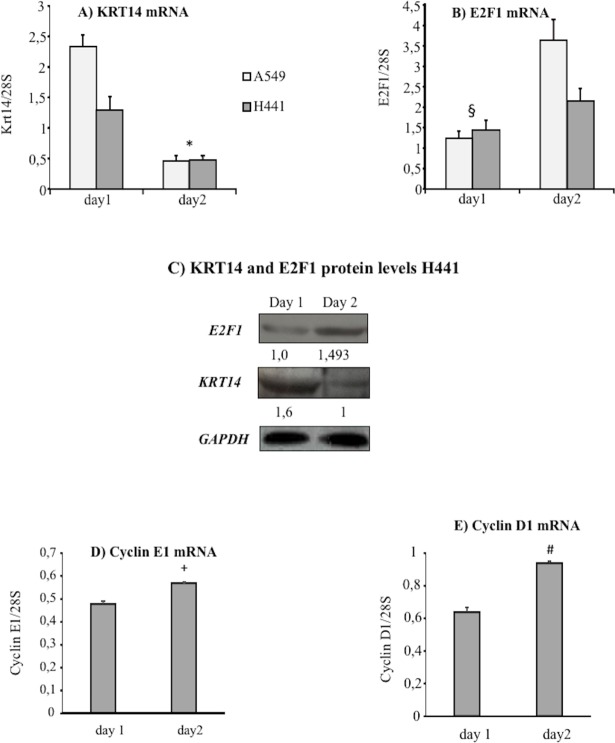
Kinetic of KRT14, E2F1, cyclinE1 and cyclin D1 expression in human pulmonary cell lines A549 and H441. A) KRT14 mRNA levels in A549 and H441 lines at day 1 and 2 under basal growth conditions; n = 6, *p<0,05 compared to day 1, data were normalized to the levels of 28S. B) E2F1 mRNA level in A549 and H441 lines at day 1 and 2 under basal growth conditions, n = 6, § p<0.05 compared to day 2, data were normalized to the levels of 28S. C) Protein level of KRT14 and E2F1 in H441 assessed by Western blot at day 1 and 2 under basal growth conditions; GAPDH level was used for normalization. D) Cyclin E1 mRNA expression level in H441 at day 1 and 2 under basal growth conditions, n = 5, + p<0.001 compared to day 2, data were normalized to the levels of 28S. E) Cyclin D1 mRNA level in H441 at day 1 and 2 under basal growth conditions, n = 5, # p<0.001 compared to day 2, data were normalized to the levels of 28S. All data are reported as mean ± SD.

**Fig 5:**
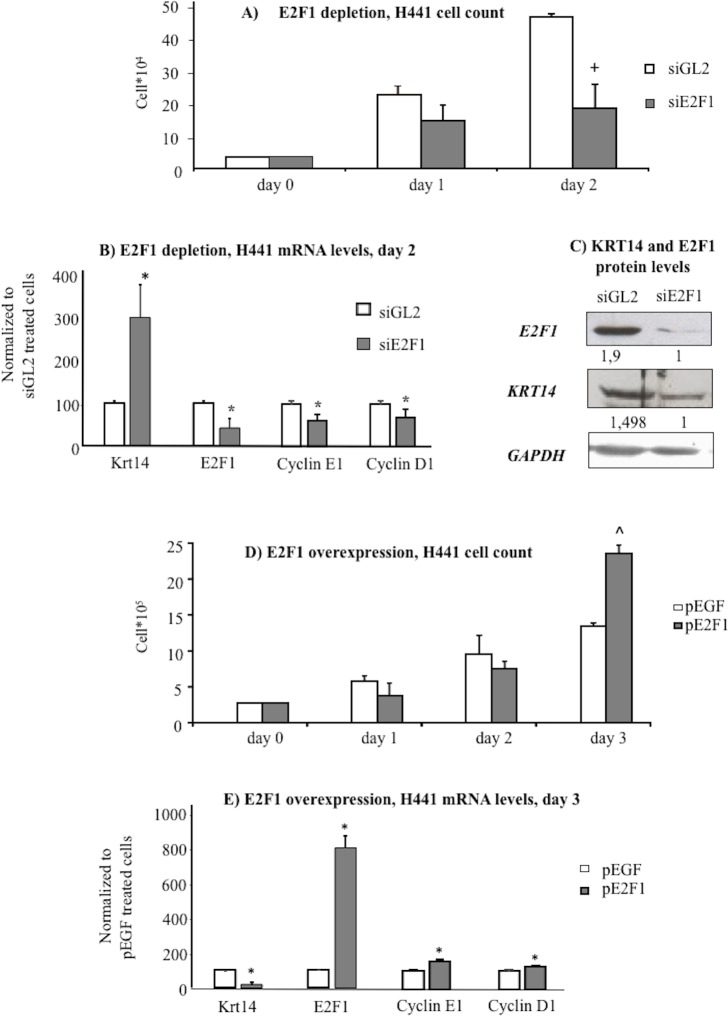
Effects of E2F1 depletion/overexpression in the human pulmonary cell line H441. A) Cell count at day 0,1,2, and 3 after E2F1 depletion by siRNA (siE2F1) compared with control siRNA (siGL2), n = 4, +p<0,05 compared to siGL2. B) mRNA levels of KRT14, E2F1, cyclin E1 and cyclin D1 at day 2 after cell cycle arrest caused by E2F1 depletion (siE2F1), n = 6, *p<0.05 compared to siGL2. C) Protein levels of KRT14 and E2F1 in H441 assessed by Western blot at day 2 following E2F1 depletion by siE2F1; GAPDH level was used for normalization. D) Cell count at day 0,1,2, and 3 after E2F1 overexpression, n = 3, ^p<0.05 compared to pEGFP. E) mRNA expression levels of KRT14, E2F1, cyclin E1 and cyclin D1 at day 3 after cell cycle progression induced by E2F1 overexpression (pE2F1) compared with an unrelated transfection vector (pEGFP), n = 4, *p<0.05 compared to pEGFP. All data are reported as mean ± SD.

Under the culturing conditions adopted, the growth of H441 and A549 cells was optimal as highlighted by the steady increase of cell number and cell viability over time (Fig 3A and 3B). In these conditions, KRT14 mRNA and protein levels were particularly elevated one day after cell seeding, but significantly decreased two days after seeding (Fig 4A and 4C). In contrast, the levels of the proliferation marker E2F1 (Fig 4B and 4C), cyclin E1 (Fig 4D) and cyclin D1 (Fig 4E) increased from day 1 to day 2, thus showing a trend inversely related to KRT14 levels (Fig 4A and 4C). The observation that this inverse correlation between KRT14 and E2F1 occurs in two lung cell lines, suggests that the phenomenon is unlikely to represent a cell line-dependent artifact. To confirm the inverse correlation between KRT14 and E2F1, we then evaluated the effects of the siRNA-mediated depletion of E2F1 in H441 cells also considering the other two proliferation markers, cyclin E1 and cyclin D1. This cell line was used because of the higher transfection efficiency compared to the A549 line. Two days following siE2F1 transfection (transfection rate 90 ± 2.7%, see S1A, S1B and S1E Fig), a significant reduction in cell growth (Fig 5A) and in E2F1, cyclin E1 and cyclin D1 levels (Fig 5B and 5C) were observed, compared to cells treated by the control siGL2 siRNA. In contrast, KRT14 levels were increased (Fig 5B and 5C).

We then evaluated the effects of E2F1 over-expression on KRT14 mRNA levels. E2F1 over-expression (transfection rate 94± 1,3%, see S1C, S1D and S1E Fig) resulted in a significant growth stimulation three days after E2F1-encoding plasmid transfection, compared to cell transfected by a control plasmid encoding the EGFP protein (Fig 5D). This time point revealed an evident increase of E2F1 mRNA levels (Fig 5E) paralleled by an increase of the other two proliferation markers cyclin E1 and cyclin D1, whose expression is related to E2F1. In contrast, a remarkable decrease in KRT14 mRNA levels (Fig 5E) was observed. Together, these results highlight the inverse relation between KRT14 and the E2F1-cyclin E1-cyclin D1 circuit.

**Fig 6:**
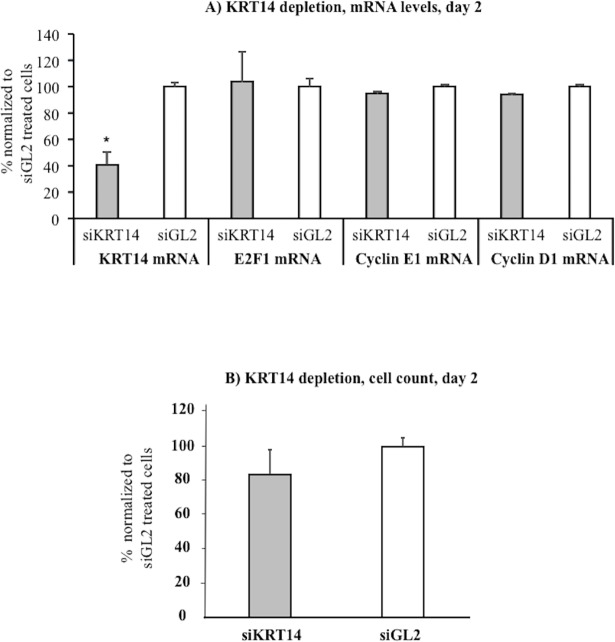
Effects of KRT14 depletion on KRT14, E2F1, cyclin E1 and cyclin D1 mRNA levels and cell growth in human pulmonary cell line H441. A) KRT14 mRNA levels following siRNA-mediated depletion (siKRT14), day two after siKRT14 transfection, n = 6, *p<0.05 compared to siGL2. E2F1, cyclin E1 and cyclin D1 mRNA levels following cell treatment by siKRT14, day two after siKRT14 transfection, n = 6. B) Cell count at day two after KRT14 depletion by siRNA (siKRT14) compared with control siRNA (siGL2). All data are reported as mean ± SD.

Finally, to explore the functional role of KRT14 in H441 proliferation its expression was downregulated by means of a specific pool of siRNAs (siKRT14). The data presented indicate that KRT14 silencing (Fig 6A) resulted in a modest reduction in H441 cell growth as evaluated by cell counting (Fig 6B). Notably, KRT14 silencing also did not affect the mRNA level of the E2F1-cyclin E1-cyclin D1 circuit (Fig 6A).

## Discussion

We have previously showed that Keratin 14 (KRT14) is overexpressed in human lung alveoli during diffuse alveolar damage (DAD), while normal lung do not [3]. DAD is a pulmonary pathology showing either features of injured collapsed alveoli and lung tissue repair/regeneration [13, 14]. The activation and proliferation of type 2 pneumocytes, a recognized progenitor cell population in human adult lung [15], may reflect both alveolar damage and regeneration [2, 16–18], but the process of KRT14 expression during pneumocytes proliferation and differentiation was not yet clarified.

To further investigate the potential role of KRT14 expression during alveolar epithelial cell proliferation and differentiation, we assessed KRT14 at transcriptional and protein levels in quiescent, proliferating and differentiating pneumocytes. We studied human lung samples with DAD and interstitial lung disease (ILD), and cultures of activated human alveolar epithelial cells (HPAEpiC). Furthermore, we carried out *in-vitro* transfection experiments on H441 and A549 cell lines for arresting or inducing the cell proliferation by silencing/stimulating the cell cycle regulatory protein-E2F1. We observed increased KRT14 mRNA and protein levels in human lung biopsies of lung repair/regeneration, but not in quiescent normal lung specimens. Of interest, it should be noted that also cultured pneumocytes showed increased KRT14 mRNA expression levels during activation of cell and before transdifferentiation (Fig 2). Further work will be required to validate this observation at the protein expression level, because it is well known that mRNA levels may not be proportional to protein levels, since the processes of translation and protein degradation are at least as important as mRNA transcription and stability to steady-state protein abundance [19]. Nevertheless, we have detected the expression of KRT14 mRNA only in DAD lung samples and increased KRT14 protein levels were found also in *in-vitro* experiments. In conclusion, all these experiments have allowed us to expand our knowledge of KRT14 expression in lung injury. It is well known that KRT14 is a key component of the intermediate cytoplasmic microfilaments which are essential for elongation, proliferation, and migration of epidermis progenitor epithelial cells in response to injury [20, 21]. The dynamic assembly and disassembly of keratin-based cytoplasmic microfilaments is needed to induce stemness in quiescent cells to foster tissue repair and regeneration [22, 23]. Keratins are also known to have a major function in providing stability to epithelial cells under conditions of mechanical stress [24]. This is exemplified by cell frailty in human inherited keratin disorders [25, 26], and in knockout mice expressing dominant-negative keratin subunits [27, 28]. In any epithelia the dynamic plasticity of keratin filaments allows cells to proliferate during renewal growth and to migrate during wound healing [29]. KRT14 null mice show extensive blistering and die 2d after birth, indicating the functional importance of KRT14 in maintaining mechanical integrity of the stratified epithelial cells [30, 31]. Moreover, KRT14 knockdown of epithelial cells leads to substantial reduction in cell proliferation [32]. Cellular KRT14 expression in epithelia is higher when the pneumocytes change shape and move [22]. However, direct correlation with pneumocyte growth has never been observed before our investigation. Our studies with pneumocyte derived cell lines indicate that KRT14 expression is maximal in the initial phase of cell proliferation and then decreases (Figs 3 and 4A) with the progression of cell growth. Remarkably, KRT14 expression is also high in the proliferating HPAEpiC (pneumocytes type 2) and decreases after plating, a process that induces transdifferentiation, as shown by the decrease in the expression of the pneumocyte type 2 marker SFTPA1 (Fig 2B). The different experimental condition adopted to evaluate KRT14 expression in pneumocyte cell lines and primary HPAEpiC depends on the very limited growth capacity of this last cell type *in-vitro*. The fact that KRT14 silencing has a modest effect on H441 growth (Fig 6) suggests that KRT14 may not have a pivotal role in the proliferation of the pneumocyte-derived cell line we considered. However, we cannot exclude that this phenomenon may depend on the kinetic of siKRT14 action. The siKRT14 used has the peak of mRNA silencing activity at day two post transfection (Fig 6A and data not shown), which corresponds to the starting point of the experiment. However, two days after plating (starting point of the experiment), KRT14 tends to drop (Fig 4A) in untreated H441. Thus, we cannot exclude that siKRT14 mostly exerts its action when KRT14 is no longer so essential for H441 growth. Notably, also at an early time point (24hrs after siKRT14 transfection), no major effects on cell growth were observed (data not shown). To fully unravelling the functional role of KRT14 in pneumocytes, future experiments performed in primary pneumocytes are necessary.

Despite the uncertainty about the functional role of KRT14 in pneumocytes proliferation, our data collectively point towards establishing the role of KRT14 as a marker of early proliferation in both cell types, strengthening our previous findings *in-vivo* [3], where we showed that KRT14 immunoreactivity was mainly expressed in hyperplastic type 2 pneumocytes of lungs having pathologic features of lung regeneration/repair [3]. Although features of wound-repair/regeneration are common in DAD [33], it is not surprising that it can be also found in other pulmonary diseases, like some ILD [34]. Our current findings confirm the expression of KRT14 in most cases of ARDS-linked DAD (both at mRNA and protein levels), but also in some patients with ILD having an ongoing repair process.

The expression of KRT14 during our *in-vitro* experiments inversely correlated with the proliferative marker E2F1, cyclinE1 and cyclin D1 (Figs 4 and 5B–5E), similarly to our previous observation done in human DAD specimens [3]. Moreover, in our previous work, we observed that most KRT14+ pneumocytes were also Ki67-, another well-known proliferation marker. Therefore, both alveolar epithelial cells during *in-vitro* growth and alveolar cells of human DAD specimens show identical behavior, suggesting that our observations are consistent and significant. Very recently, Eickelberg’group found similar results in distal airway epithelial progenitor cells [35]. Both Eickelberg’s and our study may throw a new light on the key role of KRT14 in the pathobiology of lung injury-linked diseases: this keratin is not expressed in airways and alveoli during progenitor cells quiescence, but it is expressed during the initial phases of repair/regeneration process. We did not explore the functional role of Keratin14 in experimental models of fibrotic lung disease. Nevertheless, while several experimental models failed to fit the real human disease [36], our results were obtained in human samples and *in-vitro* human cell populations, so representing a convincing and intriguing proof of concept for further experimental researches.

In conclusion, the results of our study suggest that KRT14 can be considered a viable biomarker of early lung regenerative/disease processes. Further studies on the progenitor properties of type 2 pneumocytes could lead to the discovery of novel therapeutic approaches for the treatment of acute and chronic lung diseases.

## Supporting information

S1 Fig Transfection efficiency of FITC-labelled siRNA GL2 and of pEGFP. At the end of transfection by FITC-labelled siGL2 (220 nM), FITC labelled cells were photographed (A-B) and the amount (E) quantified by flow cytometry (data are expressed as mean ± SD, n = 4). 24 hours after transfection by pEGFP, EGFP labelled cells were photographed (C-D) and the amount (E) quantified by flow cytometry (data are expressed as mean ± SD, n = 4). Overexpression of E2F1 by pE2FP is shown by western blotting (F) in comparison to non transfected cells (NT).
(PDF)
