# Supplementary material for: Keratin14 mRNA expression in human pneumocytes during quiescence, repair and disease
Source: PLoS One. 2017 Feb 15;12(2):e0172130. doi: 10.1371/journal.pone.0172130 (PMC5310884; doi:10.1371/journal.pone.0172130)
Supplement: S1 Fig — At the end of transfection by FITC-labelled siGL2 (220 nM), FITC labelled cells were photographed (A-B) and the amount (E) quantified by flow cytometry (data are expressed as mean ± SD, n = 4). 24 hours after transfection by pEGFP, EGFP labelled cells were photographed (C-D) and the amount (E) quantified by flow cytometry (data are expressed as mean ± SD, n = 4). Overexpression of E2F1 by pE2FP is shown by western blotting (F) in comparison to non transfected cells (NT). (PDF) [file pone.0172130.s001.pdf]

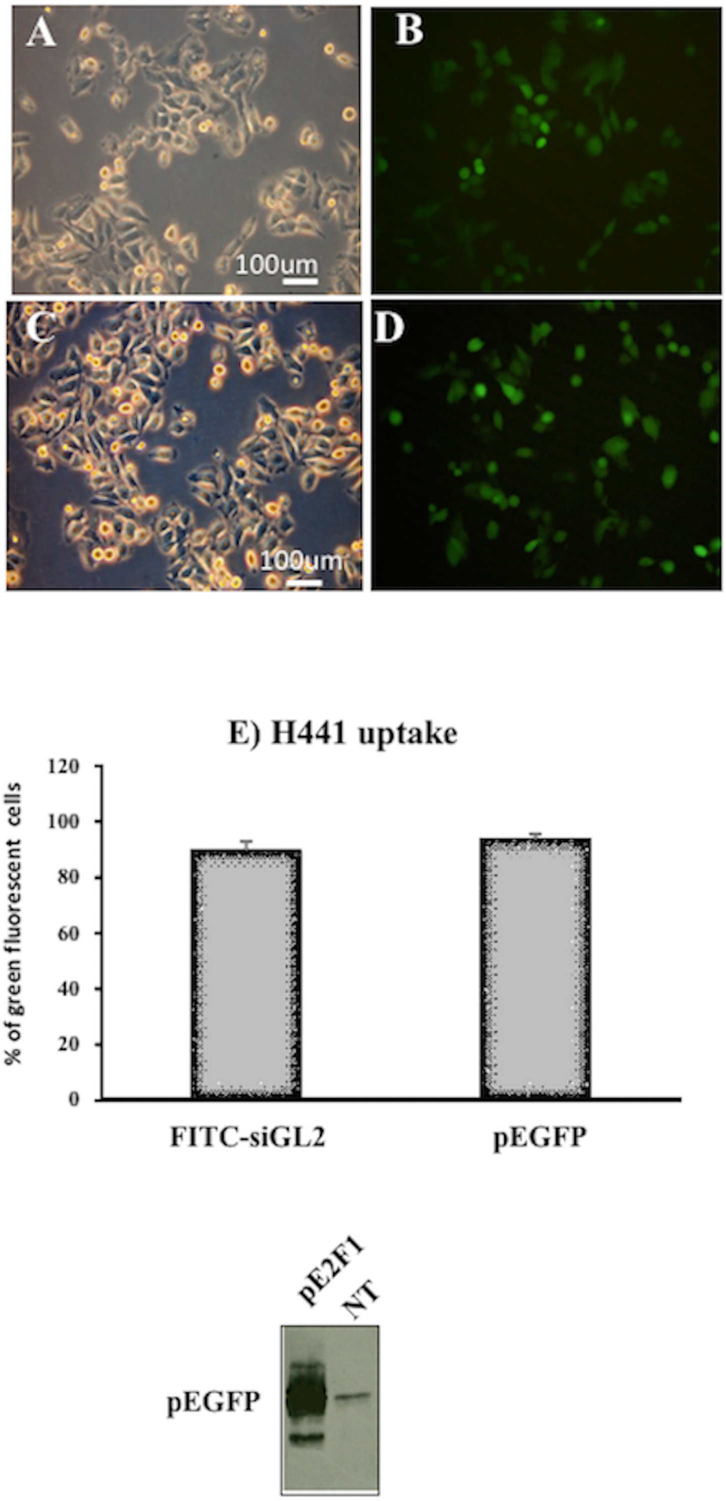

Transfection efficiency FITC-labelled siRNA GL2 and of pEGFP . At the end of transfection by FITC-labeled siGL2 (220 nM), FITC labelled cells were photographed (A-B) and the amount (E) quantified by flow cytometry (data are expressed as mean ± SD, n = 4). 24 hours after transfection by pEGFP, EGFP labelled cells were photographed (C-D) and the amount (E) quantified by flow cytometry (data are expressed as mean ± SD, n = 4). Overexpression of E2F1 by pE2F1 is shown by western blotting (F) in comparison to non transfected cells (NT).
